# Facing emotions: real-time fMRI-based neurofeedback using dynamic emotional faces to modulate amygdala activity

**DOI:** 10.3389/fnins.2023.1286665

**Published:** 2024-01-11

**Authors:** Apurva Watve, Amelie Haugg, Nada Frei, Yury Koush, David Willinger, Annette Beatrix Bruehl, Philipp Stämpfli, Frank Scharnowski, Ronald Sladky

**Affiliations:** ^1^Department of Psychiatry, Psychotherapy, and Psychosomatics, Psychiatric University Hospital, University of Zürich, Zürich, Switzerland; ^2^Department of Child and Adolescent Psychiatry, Psychiatric Hospital, University of Zürich, Zürich, Switzerland; ^3^Magnetic Resonance Research Center (MRRC), Department of Radiology and Biomedical Imaging, Yale University, New Haven, CT, United States; ^4^Division of Psychodynamics, Department of Psychology and Psychodynamics, Karl Landsteiner University of Health Sciences, Krems an der Donau, Lower Austria, Austria; ^5^Neuroscience Center Zürich, University of Zürich and Swiss Federal Institute of Technology, Zürich, Switzerland; ^6^Center for Affective, Stress and Sleep Disorders, Psychiatric University Hospital Basel, Basel, Switzerland; ^7^Zurich Center for Integrative Human Physiology, Faculty of Medicine, University of Zürich, Zürich, Switzerland; ^8^Department of Cognition, Emotion, and Methods in Psychology, Faculty of Psychology, University of Vienna, Vienna, Austria; ^9^Social, Cognitive and Affective Neuroscience Unit, Department of Basic Psychological Research and Research Methods, Faculty of Psychology, University of Vienna, Vienna, Austria

**Keywords:** real-time fMRI, neurofeedback, amygdala, emotion regulation, dynamic faces

## Abstract

**Introduction:**

Maladaptive functioning of the amygdala has been associated with impaired emotion regulation in affective disorders. Recent advances in real-time fMRI neurofeedback have successfully demonstrated the modulation of amygdala activity in healthy and psychiatric populations. In contrast to an abstract feedback representation applied in standard neurofeedback designs, we proposed a novel neurofeedback paradigm using naturalistic stimuli like human emotional faces as the feedback display where change in the facial expression intensity (from neutral to happy or from fearful to neutral) was coupled with the participant’s ongoing bilateral amygdala activity.

**Methods:**

The feasibility of this experimental approach was tested on 64 healthy participants who completed a single training session with four neurofeedback runs. Participants were assigned to one of the four experimental groups (*n* = 16 per group), i.e., happy-up, happy-down, fear-up, fear-down. Depending on the group assignment, they were either instructed to “try to make the face happier” by upregulating (happy-up) or downregulating (happy-down) the amygdala or to “try to make the face less fearful” by upregulating (fear-up) or downregulating (fear-down) the amygdala feedback signal.

**Results:**

Linear mixed effect analyses revealed significant amygdala activity changes in the fear condition, specifically in the fear-down group with significant amygdala downregulation in the last two neurofeedback runs as compared to the first run. The happy-up and happy-down groups did not show significant amygdala activity changes over four runs. We did not observe significant improvement in the questionnaire scores and subsequent behavior. Furthermore, task-dependent effective connectivity changes between the amygdala, fusiform face area (FFA), and the medial orbitofrontal cortex (mOFC) were examined using dynamic causal modeling. The effective connectivity between FFA and the amygdala was significantly increased in the happy-up group (facilitatory effect) and decreased in the fear-down group. Notably, the amygdala was downregulated through an inhibitory mechanism mediated by mOFC during the first training run.

**Discussion:**

In this feasibility study, we intended to address key neurofeedback processes like naturalistic facial stimuli, participant engagement in the task, bidirectional regulation, task congruence, and their influence on learning success. It demonstrated that such a versatile emotional face feedback paradigm can be tailored to target biased emotion processing in affective disorders.

## Introduction

Worldwide, nearly a billion people suffer from mental health problems in some form of mood disorder that significantly affects their quality of life (World Health Organization, 2022). Among the most common mood disorders are affective disorders, such as major depressive disorder (MDD), and anxiety disorder (AD), which have the highest global prevalence rate and cause a social and economic burden (Steel et al., 2014). Epidemiological data from Europe suggest that AD (14.0%) has the highest 12-month prevalence rate with MDD (6.9%) in the third position (Wittchen et al., 2011). They have become even more prominent with the emergence of the COVID-19 pandemic in 2020 (Santomauro et al., 2021). In addition to disrupting emotional well-being and behavior, affective disorders also affect somatic health (Momen et al., 2020), which can lead to reduced life expectancy (Nordentoft et al., 2013). They are substantially associated with high treatment costs, disability, and chronicity (Cuijpers et al., 2012; Baxter et al., 2014). Considerable financial and scientific resources have been invested in the development of efficient and cost-effective treatments for MDD and AD in the fields of neuropsychopharmacology, brain stimulation, and psychotherapy. However, further research into different treatment approaches for the improvement of AD and MDD symptoms is warranted due to the significant number of non-responders.

In terms of symptomatology, affective disorders are mainly characterized by affective biases, dysfunctional self-belief (Hofmann et al., 2012), and deficits in emotion regulation (Joormann and Siemer, 2014). Established research suggests that the maladaptive functioning of cortico-limbic regions, particularly the amygdala, is involved in the development and propagation of these symptoms (Disner et al., 2011). Altered amygdala responses to affective stimuli trigger emotion dysregulation, manifesting hallmark MDD symptoms such as anhedonia, i.e., diminished positive affectivity (McMakin et al., 2012), and amplified negative emotional responses, resulting in dysphoria, i.e., feelings of fear, anxiety, distress, etc. (Siegle et al., 2007). Neural signatures of MDD reveal that these affective biases are associated with decreased amygdala activity in response to positive stimuli and increased reactivity to negative stimuli (Suslow et al., 2010; Victor et al., 2010; Young et al., 2016). Reduced amygdala reactivity to happy faces in MDD patients correlates with higher anhedonia scores and poorer or inappropriate salience attributions to positive environmental cues (Victor et al., 2010). Such impaired processing of positive affect is also associated with an increased bias toward sad faces, which correlates with MDD severity (Suslow et al., 2010).

In anxiety disorders such as posttraumatic stress disorder (PTSD) and social anxiety disorder (SAD), attentional and emotion-processing biases lead to exaggerated responses to negative stimuli that are perceived as threatening. Fear is one of the core symptoms of anxiety disorders, e.g., fear of negative social evaluation and speech anxiety in SAD (Tillfors et al., 2002; Davies et al., 2017). Studies suggest that hyperactivation of the amygdala is associated with the biased processing of negative emotions that trigger fear responses in individuals with AD. There is evidence that amygdala activity scales with the intensity of emotional images (Karlsson et al., 2010) and faces (Wang et al., 2017). Amygdala hyperactivity has been observed in response to negative (Etkin and Wager, 2007) and neutral facial expressions in AD (Cooney et al., 2006). For example, patients suffering from SAD showed increased amygdala activity compared to healthy controls when presented with emotional faces (Stein et al., 2002; Phan et al., 2006; Schneier et al., 2009).

These findings of impaired neural processing of affective stimuli, such as emotional faces, are replicated in depression and anxiety disorders (Stein et al., 2002; Campbell et al., 2007; Etkin and Wager, 2007). Although the amygdala is not the only brain region relevant to the perception and regulation of mood and emotion (Pessoa and Adolphs, 2010; Grupe and Nitschke, 2013), its critical involvement in these processes remains undisputed. Current empirical evidence confirms that the amygdala not only plays a critical role in the detection and processing of emotionally salient stimuli but is also involved in emotional memory formation, fear conditioning, social cognition, and reward processing (Bickart et al., 2011; Inman et al., 2020; Domínguez-Borràs and Vuilleumier, 2022). These neural mechanisms are necessary for appropriately processing emotional cues and subsequent behavioral responses. Thus, the ability to regulate amygdala responses may indicate successful context-dependent affect processing. Unfortunately, its location deep within the temporal lobe limits the applicability of non-invasive exogenous brain stimulation methods such as transcranial magnetic and electrical stimulation. Therefore, a new form of non-invasive and individualized treatment is needed to address the heterogeneous symptoms of affective disorders.

The application of a novel form of endogenous brain stimulation method, such as real-time functional magnetic resonance imaging-based neurofeedback (rt-fMRI NF), could be a promising alternative or adjunct to current treatment regimens for affective disorders. Using this technique, participants can learn to voluntarily control their neural responses by modulating their brain activity, which is presented in the form of a real-time feedback signal. Previous rtfMRI-NF studies of amygdala self-regulation have already demonstrated the benefits of this new non-invasive approach in improving emotion regulation in healthy individuals and various psychiatric disorders (e.g., Linden et al., 2012; Brühl et al., 2014; Zotev et al., 2016; Young et al., 2017; Koush et al., 2017b; Paret et al., 2018; Zaehringer et al., 2020).

However, these previous studies used a symbolic representation of the feedback signal, such as a thermometer-like scale. Here, we investigated the applicability of a novel feedback approach using adaptive naturalistic face stimuli. Instead of a discrete and abstract representation of the neurofeedback signal, we used a sequence of human faces displaying smooth transformations of varying intensities of neutral to happy or neutral to fearful facial emotions that were generated using face-morphing algorithms. This novel feedback modality might have several advantages over conventional feedback. First, social feedback is highly relevant because it reflects interactions that humans encounter in their natural habitat, and thus emotional faces are more ecologically valid than abstract symbolic feedback representations (Mathiak et al., 2015). Second, previous studies simultaneously presented the visual cue and the feedback signal at different locations or subsequently, in order to modulate stimulus-induced activity through neurofeedback training (Brühl et al., 2014; Paret et al., 2014; Hartwell et al., 2016). Such dual-task interference between stimulus perception and feedback monitoring can be avoided by combining amygdala-relevant pictorial cues (Geissberger et al., 2020) and the feedback signal. Third, interactive emotional faces (i.e., a fearful face that gradually becomes less fearful, and a neutral face that becomes happier) are more socially motivating as the participant’s regulation effort has real motivational consequences in terms of approach and avoidance of the task-relevant feedback signal (Carver, 2006; Ihssen et al., 2017).

A few neurofeedback studies have used interactive feedback interfaces such as human avatars that dynamically change emotional expressions corresponding to the targeted neural activity (Mathiak et al., 2015; Direito et al., 2019, 2021). The current study paradigm included a more realistic and dynamic feedback presentation of human face stimuli. We argue that this type of innovative feedback could be more naturalistic, ecologically valid, and socially rewarding as compared to virtual avatars (Philip et al., 2018). Finally, this setup also allows for more complex research designs inspired by closed-loop control theory to shape brain dynamics through positive (i.e., a strong brain response causes a stronger stimulus) and negative (i.e., a strong brain response causes a weaker stimulus) feedback loops that increase or decrease neural responsivity, respectively (Toates, 1975; Mulholland and Eberlin, 1977; Mulholland et al., 1979; Pope et al., 1995).

**Figure 1:**
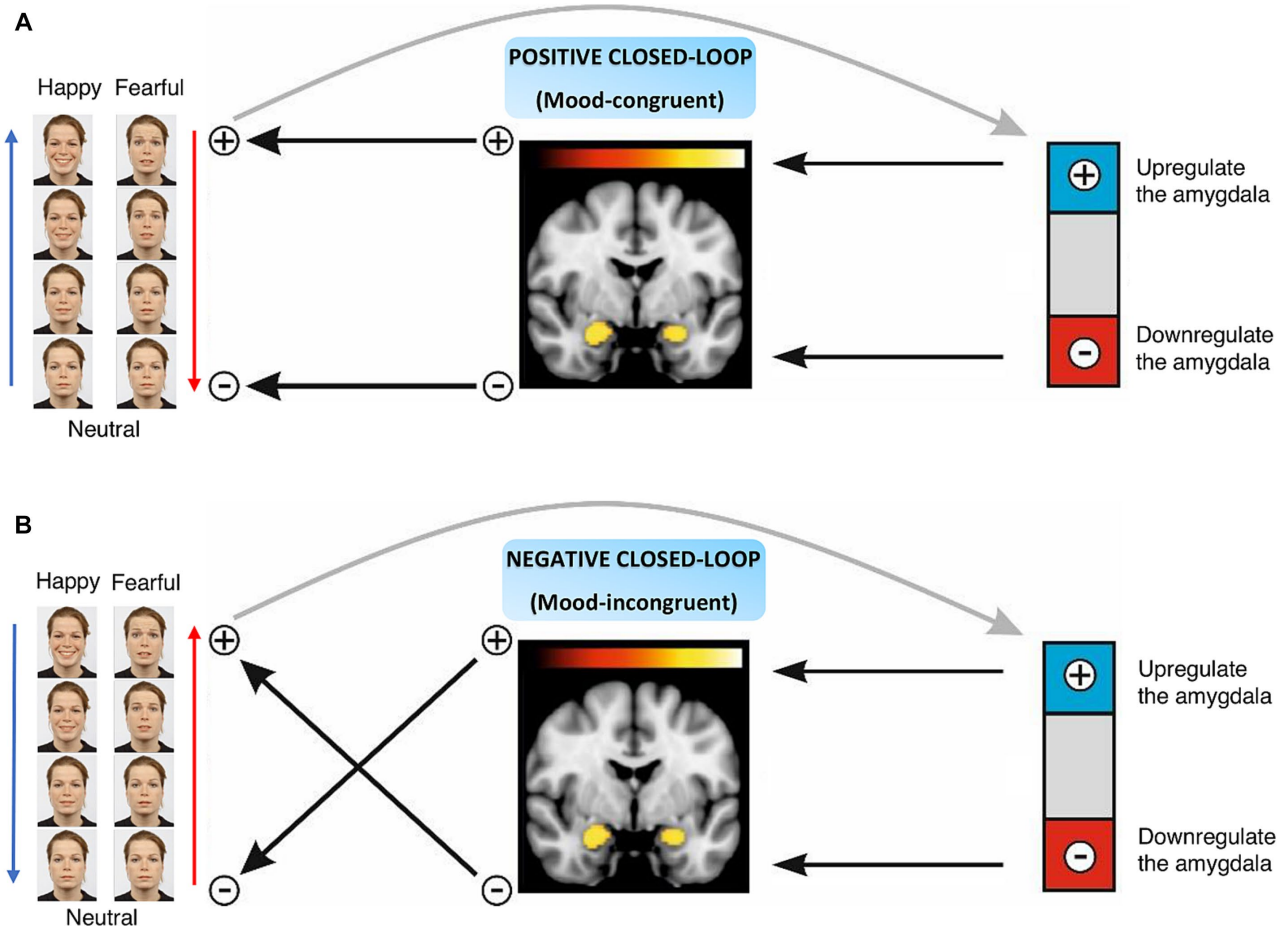
Naturalistic closed-loop neurofeedback design. **(A)** Positive closed-loop includes task-congruent conditions, i.e., happy-up and fear-down groups, where an increase in the amygdala activity enhances the intensity of the facial affect. **(B)** Negative closed-loop characterizes task-incongruent conditions, i.e., happy-down and fear-up groups where an increase in the amygdala activity reduces the intensity of the facial affect and vice versa.

In this study, we tested the efficacy of this novel closed-loop neurofeedback approach in healthy participants. The prospective goal is to use this tailored adaptive experimental therapy to target affective biases and associated symptoms in psychiatric disorders. Participants were randomly assigned to four groups based on the emotional face stimuli and task congruency. Participants were instructed to make the neutral face happier by increasing their amygdala activity in the happy-up (task-congruent) and decreasing it in the happy-down (task-incongruent) groups. Whereas participants were instructed to reduce the fearfulness of the face by downregulating their amygdala activity in the fear-down (task-congruent) group and upregulating it in the fear-up (task-incongruent) group, respectively (Figure 1). Perceiving facial expressions can help regulate emotional responses, such as enhancing positive mood or reducing negative affective state, especially when the expression of the face stimulus matches one’s intentions, in other words, is congruent with one’s regulatory efforts. For example, in the happy-up (task-congruent) group, the task goal was to increase the happiness of the face stimulus by upregulating amygdala activity, i.e., the face stimulus is rewarding the inherent human tendency to perceive happy faces by upregulating the amygdala. On the other hand, being exposed to emotional stimuli that are not in alignment with one’s regulatory efforts can lead to task incongruency. For example, in the happy-down group, the goal was to increase the happiness of the face stimulus by decreasing amygdala activity. In this condition, the rewarding face stimuli (happy faces) contradict the task goal of downregulating the amygdala. This type of feedback setup may require greater cognitive effort due to internal conflict of pursuing a reward by engaging contrasting neural patterns and may provide a training opportunity akin to exposure therapy. With such a versatile and comprehensive feedback design, we hypothesized that healthy participants would learn to upregulate and downregulate their amygdala activity in the task-congruent groups, i.e., happy-up and fear-down, respectively, as compared to the respective task-incongruent groups (happy-down and fear-up).

Next, we hypothesized that the task-dependent effective connectivity between the amygdala and the prefrontal cortex and/or face-sensitive regions in the temporal lobe would increase over the course of the neurofeedback training session. In the context of affective disorders, maladaptive emotional responses are manifested due to dysfunctional inhibitory control of higher order cognitive top-down processes on the maladaptive bottom-up pathways regulated by subcortical regions like the amygdala (Nicholson et al., 2017). Previous work has already demonstrated the role of the fusiform face area (FFA) and the medial orbitofrontal cortex (mOFC) in face processing and amygdala regulation (Kanwisher et al., 1997; Almeida et al., 2009; de Almeida et al., 2011; Sabatinelli et al., 2011; Roy et al., 2012; Sladky et al., 2015). We employed dynamic causal modeling (DCM; Friston et al., 2003), a Bayesian framework, to assess the directionality of neural dynamics involved in emotion processing (Krylova et al., 2021; Sladky et al., 2022). Specifically, we hypothesized that upregulation of the amygdala would result in positive (facilitatory), and downregulation would entail negative (inhibitory) connectivity between the mOFC and the amygdala. Finally, we assessed the intervention-induced mood changes in the participants using self-rated psychometric questionnaires such as the Positive And Negative Affect Schedule (PANAS, Watson et al., 1988) and the Self-rating Depression Scale (SDS, Zung, 1965). We hypothesized that the naturalistic closed-loop training would improve participants’ positive affectivity scores on the PANAS scale while decreasing PANAS negative affectivity scores, and SDS scores post-training, specifically, in the task-congruent groups (happy-up and fear-down).

## Methods

### Participants

**Figure 2:**
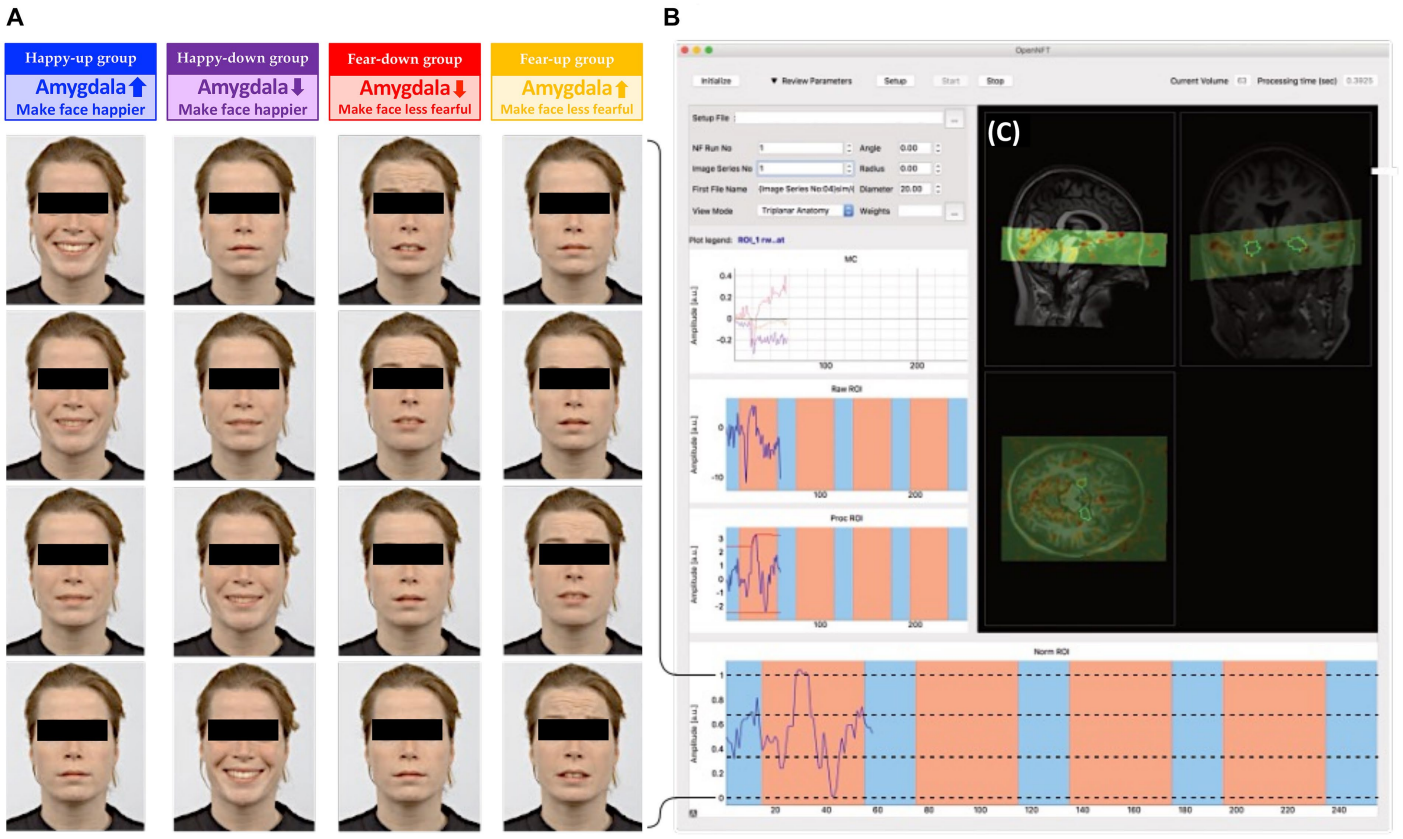
**(A)** Dynamic emotional face stimuli: Faces were digitally morphed in 30 steps from neutral to happy (happy groups) or neutral to fearful (fear groups). Subjects were instructed to either upregulate (up groups) or downregulate (down groups) the amygdala, and thus change the valence of the faces according to the group assignment. **(B)** OpenNFT display: Feedback from bilateral amygdala was continuously estimated using OpenNFT, an open-source framework for neurofeedback training (http://www.opennft.org, Koush et al., 2017a). **(C)** The field of view (green) projected on a participant’s structural scan covered the mOFC, temporal lobe (amygdala, fusiform gyrus) and parts of the visual cortex. The stimulus intensities were calculated based on an anatomical mask of the bilateral amygdala (Tyszka and Pauli, 2016), and were presented as feedback using PsychoPy.

Sixty-four healthy adults between 18 and 65 years of age, fluent in German, right-handed with normal vision and without any MRI contraindications such as pregnancy, claustrophobia, metallic implants, clinically significant somatic diseases, brain surgery, neurological disorders, and substance abuse, were recruited for the study. The participants (mean age = 25.07 ± 4.46 years) were assigned to one of the four age- and gender-matched neurofeedback intervention groups with 16 participants in each group, i.e., happy-up (mean age = 26.59 ± 4.87 years, gender = 8 m:8f), happy-down (mean age = 25.93 ± 5.93 years, gender = 8 m:8f), fear-up (mean age = 23.85 ± 3.54 years, gender = 7 m:9f), and fear-down (mean age = 23.63 ± 2.85 years, gender = 8 m:8f). They were instructed to either up or downregulate the amygdala neurofeedback signal while they were presented with neutral to happy or neutral to fearful human faces (Figure 2). Participants were asked to abstain from psychotropic substances for a minimum of 3 days and from alcohol for at least 24 h prior and nicotine/caffeine 1 h prior to the imaging session. All participants provided written informed consent in accordance with the Declaration of Helsinki and were compensated for their participation. The study was approved by the local ethics committee of the canton Zurich.

### Neurofeedback task

The neurofeedback session consisted of four training runs with each neurofeedback run having four 40-s regulation blocks interleaved with four 20-s baseline blocks, during which participants were instructed to mentally count slowly (starting from one in increments of one) while fixating on a dot. During each regulation block, a new face from the Radboud Faces Database (Langner et al., 2010) was presented continuously. The faces were randomized, unique (i.e., novel for the participant), and counterbalanced for gender. Based on the group assignment, the faces expressed some degree of positive (i.e., happy) or negative (i.e., fearful) affective state. The intensity of the emotional expression was proportional to the mean blood oxygenation level dependent (BOLD) signal intensity of the participant’s bilateral amygdala. The varying degrees of emotional intensity were achieved by smoothly blending faces with different emotional intensity using a face morphing software written in Python^1^ (Willinger et al., 2019). Thus, participants were presented with highly naturalistic face stimuli that dynamically changed depending on their amygdala activity (Figure 2). Depending on the emotional stimulus and regulation condition, participants were assigned to one of the four experimental groups and received group-specific instructions as described below:

1. happy-up: “Try to make the face happier by up-regulating the amygdala.”
2. happy-down: “Try to make the face happier by down-regulating the amygdala.”
3. fear-down: “Try to make the face less fearful by down-regulating the amygdala.”
4. fear-up: “Try to make the face less fearful by up-regulating the amygdala.”

For all groups, ongoing bilateral amygdala activity was translated into facial expression intensity either from neutral to happy (first and second groups), or from fearful to neutral (third and fourth groups), as depicted in Figure 2. Participants were informed about the amygdala’s involvement in affect processing and that the change in the expression of the face stimulus was based on their amygdala activity. They were given the freedom to use the emotion regulation strategies of their choice and to modify the strategies between runs. They were also asked to verbalize the experimental mental strategy beforehand and to remain focused during the task. Repeated sampling of participants’ feedback between each run also ensured that participants remained engaged in the task.

### Naturalistic face feedback

The naturalistic face stimuli consisted of human faces of 30 Caucasian models (15 females) from the Radboud Face Database depicting neutral, fearful, and happy emotions (Langner et al., 2010). A face morphing algorithm developed in Python was used to create dynamic emotional faces with gradually changing facial expressions in 30 steps,^2^ such that 0 corresponded to the lowest valence or neutral emotion (0%) and 30 to the highest valence (100%), i.e., fearful or happy (smiling) expressions. The intensity of the emotional valence was coupled to the average BOLD signal of the participant’s bilateral amygdalae.

The estimation and presentation of the feedback signal was achieved using the Open NeuroFeedback Training (OpenNFT) software, an open-source neurofeedback framework implemented using Python and Matlab (Koush et al., 2017a). The feedback was scaled to the normalized amygdala time-series using OpenNFT’s default dynamic range to estimate the maximum and minimum limits of the scaling (Koush et al., 2017a) by using the average of the 5% highest and lowest signal intensities observed so far. The preprocessed amygdala signal was then mapped to the intensity of the emotional expression ranging between the lowest and the highest valence of the face stimulus that served as the feedback signal.

### Region of Interest (ROI)

A high spatial resolution, three-dimensional, probabilistic *in vivo* anatomical mask of the bilateral amygdala used in the current study was based on the California Institute of Technology (CIT168) human brain templates (Tyszka and Pauli, 2016), and was co-registered non-linearly in individual MNI space (Figure 2C). The template mask was created using the SPM normalization function through inverse warping.

### Brain image acquisition

MRI acquisitions were performed using an Achieva 3-Tesla MRI scanner (Philipps Healthcare, Best, The Netherlands) and the manufacturer’s 32 channel head coil at the MR Centre of the Psychiatric Hospital, University of Zurich. We acquired 265 volumes covering the mOFC, temporal lobe (amygdala, fusiform gyrus) and parts of the visual cortex per training run using a gradient echo T2*-weighted echo planar imaging (EPI) sequence with the following parameters: TR = 1,000 ms, TE = 35 ms, 15 interleaved ascending axial slices, 2 × 2 × 2 mm^3^ voxel size, 1 mm slice gap, 112 × 110 matrix, field of view (FOV) = 224 × 224 × 44 mm^3^, flip angle of 65°, SENSE factor 2. The first five volumes were discarded as dummy scans. Additionally, a whole brain EPI volume (same parameters as above except 70 slices and a TR = 5,000 ms) and a whole brain T1-weighted structural scan (TR = 9 ms, TE = 5 ms, 160 coronal slices, 1 × 1 × 1 mm^3^ voxel size, 240 × 240 matrix, FOV = 240 × 160 × 240 mm^3^, flip angle = 8°) were acquired for online and offline data processing and to localize the bilateral amygdala. For rt-fMRI NF, brain images were exported in real-time to a high-performance computer using the proprietary Philips DRIN export system.

### Online data processing and analysis

A spatial transformation Statistical Parametric Mapping (SPM12, v7771, https://www.fil.ion.ucl.ac.uk/spm/software/spm12/) batch pipeline was used to transform the participant’s structural MRI scan, whole brain EPI volume, test EPI volume with the FOV, and a bilateral amygdala mask (Tyszka and Pauli, 2016) into the individual’s space to serve as a volume of interest for neurofeedback training. The test EPI volume was used as a reference for online realignment.

Online rt-fMRI data analysis and neurofeedback signal calculation were performed using OpenNFT (Koush et al., 2017a). OpenNFT’s default online preprocessing pipeline was used which comprised real-time realignment for motion correction and spatial smoothing (Gaussian kernel, 5 mm FWHM). Temporal data processing included spike removal using a Kalman filter, drift removal using a cumulative general linear model (GLM), a first-order autoregressive model AR (1) to account for serial correlations, and OpenNFT’s default dynamical range scaling. The extracted, preprocessed amygdala timeseries data were used to dynamically scale the intensity of the emotional expression in the faces and served as a feedback signal for the participant.

### Offline data processing and analysis

Offline data processing and analysis of the four neurofeedback runs were performed in SPM12 and comprised slice-timing correction (Sladky et al., 2013), realignment, coregistration to the participant’s whole brain EPI volume and T1-weighted structural MRI image, normalization to MNI space, and spatial smoothing with a 6 mm FWHM Gaussian kernel.

**Figure 3:**
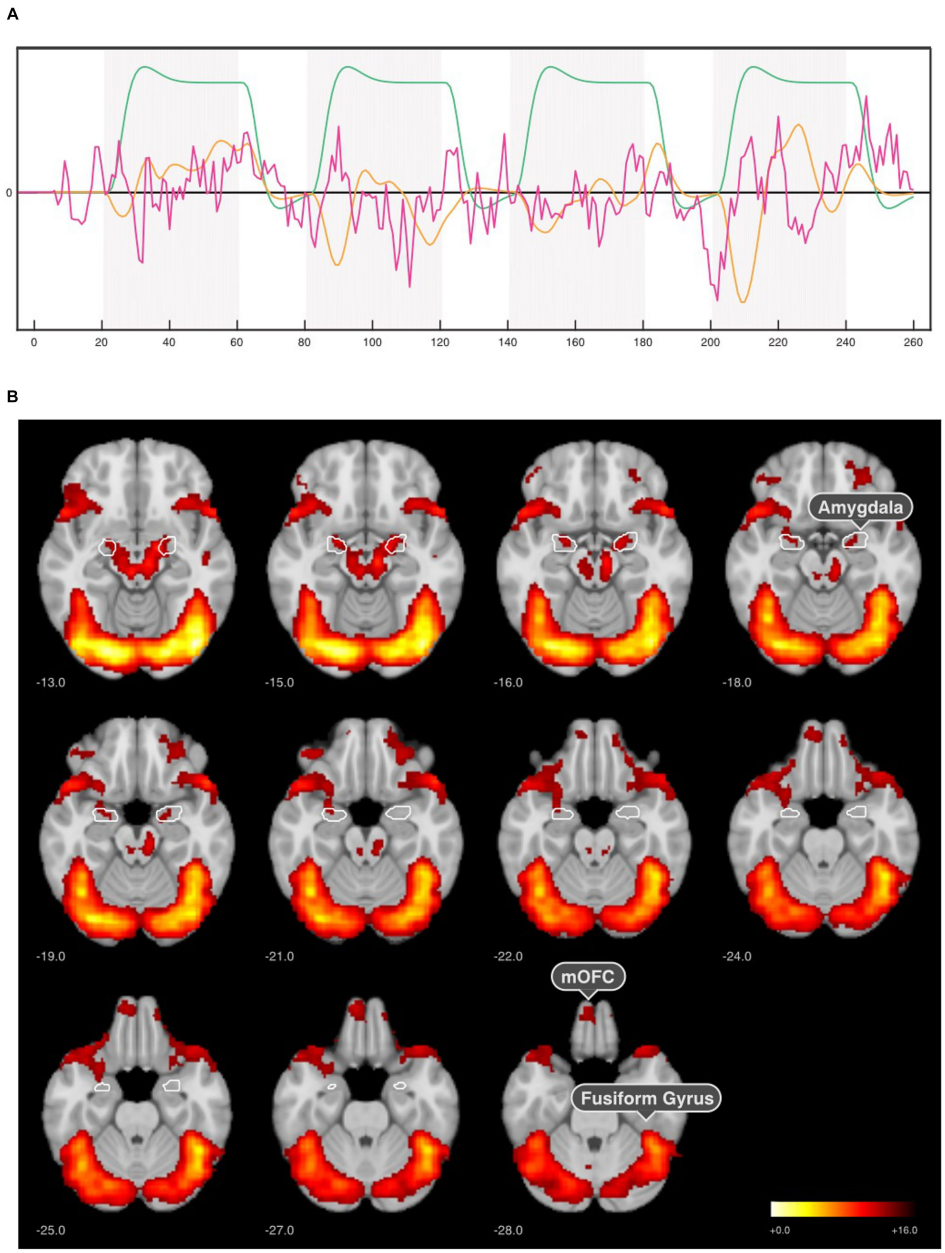
SPM-based GLM analysis results for the first neurofeedback run. **(A)** GLM model comprising three orthogonalized regressors of interest: *x_1_*—regulation blocks (green), *x_2_* -neurofeedback response (amygdala signal x cHRF, orange), and *x_3_—*neurofeedback signal (residual amygdala signal, magenta). **(B)** Regulation > Baseline contrasts for x_1_ regressor in the first neurofeedback run across all groups (*n* = 64) revealed activation in the amygdala, medial orbitofrontal cortex (mOFC), fusiform gyrus (fusiform face area). The results were corrected for *p* < 0.001 voxel-wise threshold and *p* < 0.05 cluster-level family wise error (FWE).

A subject-level analyses were performed using SPM12-based GLM only on the first neurofeedback run due to the groups’ different regulation instructions. We aimed at differentiating the individual contributions of the experimental manipulations. We expected that the task-relevant brain responses could be modeled using three orthogonalized regressors: x1 models the effects of the fMRI task condition in general, i.e., the blocks where participants were asked to regulate their amygdala response (Figure 3A, green) by convolving the box car function that encodes the regulation blocks (20s *off*, 40s *on* period) with SPM’s canonical hemodynamic response function (*cHRF*). x2 modeled the subject’s response to the emotional face that scaled proportionally to the intensity of the neurofeedback signal (Figure 3A, orange), calculated by convolving the online-extracted, normalized amygdala BOLD signal (i.e., OpenNFT’s Norm ROI signal, which was also used for neurofeedback stimulus presentation) with the *cHRF*, which corresponds to a parametric modulator of the block design. x3 was used to model the source of the neurofeedback signal (Figure 3A, magenta), which is confounded by both the regulation task (x1) and the response to the stimulus intensity (x2). To account for this, x1 and x2 were regressed out of the normalized amygdala BOLD signal, i.e., yielding a regressor (x3) that corresponds to the residual amygdala signal independent of the BOLD response to the neurofeedback stimulus intensity (x2) and regulation blocks (x1). Six realignment parameter estimates were included as nuisance regressors to account for residual movement artifacts not accounted for by the realignment preprocessing step. This design matrix captured the circularity that is inherent to the closed-loop paradigm of this study, where the stimulus intensity depends on the activity of the target region, which in turn is influenced by the stimulus intensity.

The second-level random effects group analysis consisted of a one-sample t-test of the individual contrasts between faces and baseline to identify neurofeedback-selective brain regions in the first neurofeedback run. Significance threshold for the resulting statistical parametric map was set to *p* < 0.001 (voxel-wise threshold) and *p* < 0.05 FWE cluster-level corrected.

Amygdala time-series analyses were based on OpenNFT logs from the neurofeedback experiment. We used the pre-processed amygdala signal (i.e., kalmanProc data). Each neurofeedback run consisted of four regulation blocks (dynamic face stimulus) interleaved with the four baseline blocks (a dot). Preprocessed data were modeled using GLM analysis with four regressors for the four individual regulation blocks to estimate the mean amygdala BOLD signal in the regulation condition. Contrasts were designed as a boxcar function with the 20s off (baseline) and 40s on (regulation) period convolved with SPM’s cHRF. Average values (beta weights) were extracted from the ROI beta maps associated with each regulation block and utilized in subsequent statistical analyses.

Amygdala activity changes over four neurofeedback runs were analyzed based on linear mixed-effect model (LMM) using “lme4” package (version 1.1–31, Bates et al., 2015) in R (version 4.2.0, R Core Team, 2022). LMM analysis was performed separately for the happy (happy-up and happy-down groups) and fear (fear-up and fear-down groups) conditions. For each condition, we modeled neurofeedback runs (4 runs) and groups (congruent and incongruent) categorical variables that served as fixed effects of interest with the participant as a random effect factor. For example, in the “Happy” condition, we analyzed amygdala activity changes over four runs in task-congruent (happy-up, *N* = 16) and task-incongruent (happy-down, *N* = 16) groups with a run x group interaction. The statistical model was fitted with restricted maximum likelihood (REML) using Satterthwaite’s method. A similar analysis was performed for the “Fear” condition by testing an interaction between training runs and groups (fear-down, fear-up) to assess amygdala activity changes.

### Dynamic causal modeling

Based on previous research (de Almeida et al., 2011; Sabatinelli et al., 2011; Sladky et al., 2022), we assumed that the amygdala (Amy) receives task-relevant input from the fusiform face area (FFA; Kanwisher et al., 1997) and the medial orbitofrontal cortex (mOFC; Roy et al., 2012). The primary visual cortex (V1) was considered to be the input for the experimental perturbation, i.e., the visual stimulation by emotional faces, and connected to the other regions. FFA is part of the ventral visual processing stream originating from V1 extending to the amygdala. In addition, the amygdala and mOFC also receive low-level visual input from other sources, e.g., thalamic pathways. This means in this case the direct connections from V1 do not reflect direct anatomical connectivity but a relevant task-specific, functional relationship. Omitting these connections would bias the model toward FFA-centered processing of emotional stimuli.

Dynamic causal modeling (DCM12 as implemented in SPM12, build 6,906) was used to estimate effective connectivity between task regions. Temporally filtered and detrended time courses of V1, bilateral FFA, bilateral amygdala, and medial orbitofrontal cortex were extracted for each subject using SPM’s volume of interest (VOI) extraction batch script (single-subject significance threshold *p* < 0.05, first eigenvariate used as summary statistic, adjusted for effect of interest). For the amygdala, we used the same mask that was used in the neurofeedback experiment. For the other regions, 8 mm spheres were centered around the local maximum in the group-level SPMs: V1 x/y/z = −0.0/−87.0/−6.0 mm [MNI], rFFA x/y/z = +42.0/−51.0/−24.0 mm [MNI], left FFA x/y/z = −45.0/−45.0/−25.0 mm [MNI], and mOFC x/y/z = −8.00/56.00/−26.00 mm [MNI].

The model space comprised Amy, V1, FFA, and mOFC. It was assumed *a priori* that V1 receives the driving input (i.e., modeled as the neurofeedback face regressor). Different DCM models were created where forward connections between V1 and Amy, FFA, and mOFC as well es bidirectional connections between amygdala and mOFC and FFA were considered, which resulted in 128 models. All models were estimated and analyzed using random effects Bayesian model averaging (BMA; Penny et al., 2010) to estimate the mean effective connectivity for each connection, depending on the neurofeedback run (i.e., 1–4) and group assignment (happy-up, happy-down, fear-up, fear-down).

### Clinical assessment

The influence of closed-loop amygdala neurofeedback on participants’ affectivity was investigated using self-reporting psychometric questionnaires. Participants’ positive and negative affective state changes were measured using 20-item Positive And Negative Affect Schedule (PANAS, Watson et al., 1988). The Self-rating Depression Scale (SDS, Zung, 1965) was used to screen the symptoms relating to depression. These questionnaires were filled out by the participants on a desktop computer immediately before and after their MRI measurement. Training-induced behavioral changes were assessed by fitting a LMM with PANAS and SDS scores as the outcome variable, time (pre- vs. post-training) and group (task-congruent vs. incongruent) as a fixed effect of interest, and an intercept for each participant as random effect in happy and fear conditions.

The Consensus on Reporting and Experimental Design of Clinical and Cognitive-Behavioral Neurofeedback Studies checklist (CRED-nf, Ros et al., 2020), which summarizes the current rtfMRI-NF study design, is provided in Supplementary material.

## Results

Based on SPM’s GLM analysis, the three regressors of interest were analyzed (x_1_—regulation blocks, x_2_—neurofeedback response, and x_3_—neurofeedback signal). For the face regulation blocks (x_1_), highest activation was observed in the visual cortex (occipital lobe) and fusiform face area (inferior temporal lobe) in the first neurofeedback run. The peak MNI coordinates [42, −78, −10] implied an activation in the occipital face-selective area. Besides the obvious maximum in the bilateral amygdala, this model indicated a strong BOLD response in a distributed network encompassing the mOFC, temporal, and occipital lobe in the first neurofeedback run across all participants (Figure 3B).

### Amygdala BOLD signal changes during neurofeedback training

**Figure 4:**
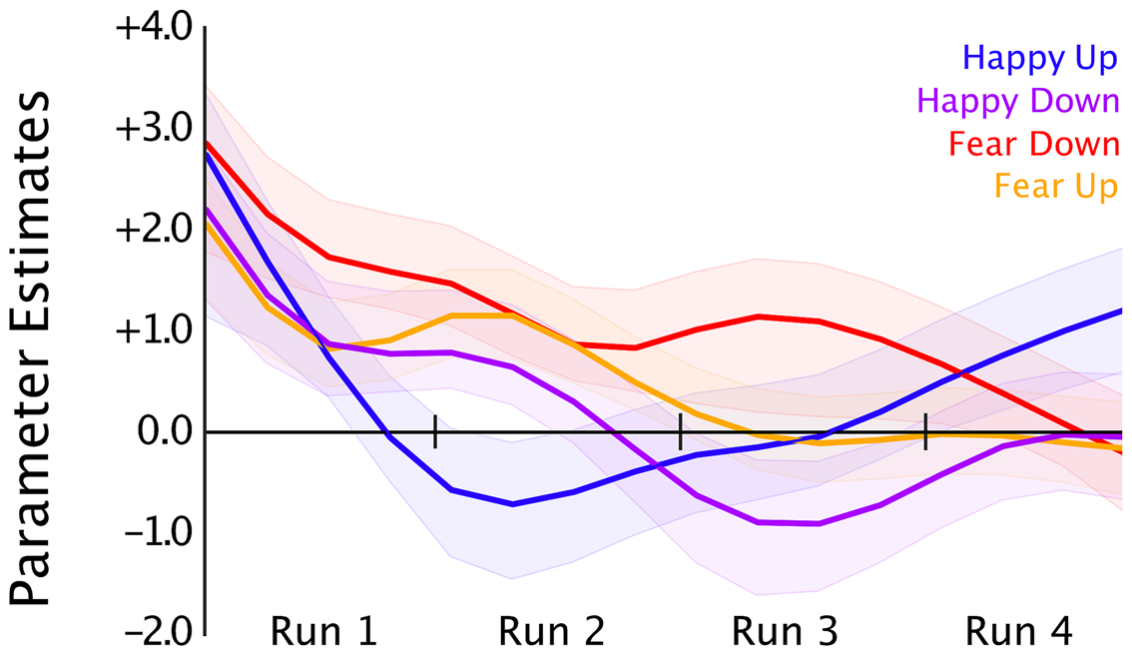
Bilateral amygdala time courses averaged (OpenNFT’s kalmanProc timeseries) over four neurofeedback training runs in the happy-up (blue, n = 16), happy-down (purple, *n* = 16), fear-down (red, *n* = 16), and fear-up (orange, n = 16) groups. OpenNFT’s filtered timeseries were modeled using a GLM with each regulation blocks as regressors, which resulted in a parameter estimate that represents the single subject mean of the block’s time course corrected for the hemodynamic delay. For display purposes, the plot of group means is smoothed using a Gaussian window (window length = 6).

Neural correlates of neurofeedback training were examined using LMM analysis by modeling run (1–4) and group (congruent and incongruent) as the fixed effects and a random intercept for participant separately in the happy and fear conditions. We observed a significant effect of run [*F*(3,90) = 5.28, *p* = 0.0002] in the fear condition with decreased amygdala activity in the third run [β = −0.34, SE = 0.11, *t*(90) = −3.04, *p* = 0.003], and the fourth run [β = −0.34, SE = 0.11, *t*(90) = −3.02, *p* = 0.003] compared to the first neurofeedback run in the fear-down group. We did not observe a significant effect of group (fear-down, fear-up) or the interaction between fixed effects, i.e., run x group. In the happy condition, LMM analyses revealed no significant effect of run, group (happy-up, happy-down), or their interaction. Amygdala activity changes over four training runs in all groups are depicted in Figure 4.

### Amygdala habituation effects within runs

Potential amygdala habituation effects within runs were investigated using a linear regression model of mean amygdala BOLD signal for each block in all participants. The resulting parameter estimates for habituation effects of each participant and run were further analyzed on a group level. No significant within-run habituation effects were observed for the happy-up and the happy-down groups. However, amygdala habituation effects were present in the last runs in the fear-down and fear-up groups. In the fear-down group, a statistically significant amygdala habituation occurred mainly in the third [Run 3: β = −0.449 ± 0.770, *t*(15) = −2.33, *p* = 0.034] and fourth runs [Run 4: β = −0.799 ± 0.853, *t*(15) = −3.75, *p* = 0.002]. Participants in the fear-up group exhibited an amygdala upregulation in the first three training runs with a significant habituation effect in the fourth run [Run 4: β = −0.622 ± 0.740, *t*(15) = −3.36, *p* = 0.004]. The results are summarized in Table 1.

**Table 1:** Estimated coefficients (β), standard errors (SE), *t* (degrees of freedom) and *p* values of the change in the amygdala activity within four training runs in four experimental groups.

Groups	NFB runs	β	SE	*t*_**(15)**_	*p*
Happy-up	1	−1.066	2.512	−1.70	0.110
	2	−0.547	1.707	−1.28	0.220
	3	−0.103	0.919	−0.45	0.659
	4	0.362	1.549	0.93	0.367
Happy-down	1	0.304	1.890	0.64	0.532
	2	0.196	0.996	0.79	0.442
	3	−0.148	0.652	−0.91	0.377
	4	−0.402	0.761	−2.11	0.052
Fear-up	1	0.559	0.889	2.52	0.024
	2	0.616^*^	0.942	2.62	0.019
	3	0.030	0.862	0.14	0.891
	4	−0.622^*^	0.740	−3.36	0.004
Fear-down	1	0.264	1.176	0.90	0.382
	2	0.088	0.907	0.39	0.702
	3	−0.449^*^	0.770	−2.33	0.034
	4	−0.799^*^	0.853	−3.75	0.002

^*^Significance at *p* < 0.05.

### Effective connectivity changes using DCM

**Figure 5:**
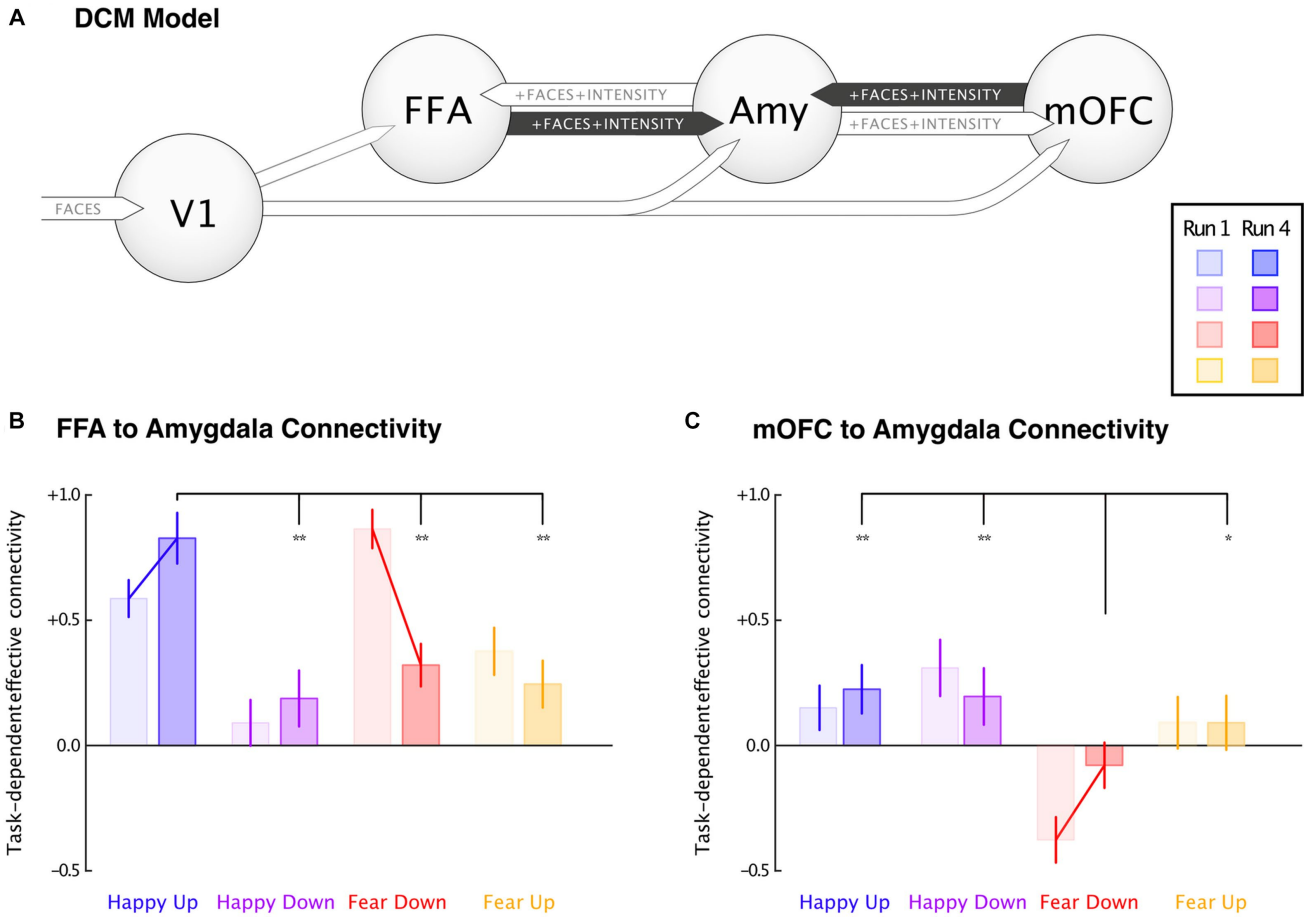
Dynamic causal modeling. Task-dependent connectivity changes were depicted between the first (light-colored bar) and the fourth (dark-colored bar) runs, and between groups. **(A)** A DCM model comprising primary visual cortex (V1), fusiform face area (FFA), medial orbitofrontal cortex (mOFC), and bilateral amygdala (Amy). **(B)** Effective connectivity changes between FFA to amygdala revealed a significant increase in the happy-up and decrease in the fear-down group, and **(C)** mOFC to amygdala connectivity showed significant negative responses in the fear-down group. Significantly decreased negative connectivity parallels reduced input from FFA. Error bars indicate 95% confidence interval, and the connecting lines denote mean significance computed using paired *t*-test. ^*^Significance at *p* < 0.05, and ^**^Significance at *p* < 0.001.

DCM Bayesian model averaging over 128 models is illustrated in Figure 5. The extrinsic inputs into V1 were similar in all groups. Positive connections were observed from V1 to FFA, Amygdala, and mOFC between groups. Task-dependent effective connectivity between FFA to amygdala and the mOFC to amygdala during the first and the fourth runs and in between group comparisons were performed using t-tests. The results revealed that participants in the happy-up group exhibited a significant task-dependent (i.e., intrinsic connectivity [A] + modulation by faces [B_1_] and the intensity of the emotion [B_2_]) upregulation of the amygdala induced by the FFA and mOFC in the last run as compared to the first run. On the contrary, the fear-down group exhibited strong upregulation of the amygdala in the first run, which is counteracted by inhibitory feedback of the mOFC. This downregulation effect persisted until the last run. While participants from happy-up group maintained the positive influence of FFA and mOFC on the amygdala over runs, connectivity in the fear-down group reduced over time. This could indicate a training-related learning mechanism in the fear-down group. Other groups did not show significant connectivity changes. The group differences in the effective connectivity between V1, FFA, amygdala and mOFC are depicted in Table 2.

**Table 2:** DCM Bayesian Model Averaging results.

VOI	Group	Run	Intrinsic [A]	Face [B_1_]	Intensity [B_2_]	Total [A + B_1_ + B_2_]
V1 → FFA	Happy-up	1	+0.17 ± 0.02^**^			+0.17 ± 0.02^**^
		4	+0.09 ± 0.02^**^			+0.09 ± 0.02^**^
	Happy-down	1	+0.03 ± 0.02^**^			+0.03 ± 0.02^**^
		4	+0.06 ± 0.02^**^			+0.06 ± 0.02^**^
	Fear-down	1	+0.16 ± 0.02^**^			+0.16 ± 0.02^**^
		4	+0.09 ± 0.02^**^			+0.09 ± 0.02^**^
	Fear-up	1	+0.14 ± 0.02^**^			+0.14 ± 0.02^**^
		4	+0.12 ± 0.02^**^			+0.12 ± 0.02^**^
V1 → Amy	Happy-up	1	+0.13 ± 0.02^**^			+0.13 ± 0.02^**^
		4	+0.04 ± 0.02^**^			+0.04 ± 0.02^**^
	Happy-down	1	+0.04 ± 0.02^**^			+0.04 ± 0.02^**^
		4	+0.01 ± 0.02			+0.01 ± 0.02
	Fear-down	1	+0.16 ± 0.02^**^			+0.16 ± 0.02^**^
		4	+0.04 ± 0.02^**^			+0.04 ± 0.02^**^
	Fear-up	1	+0.04 ± 0.02^**^			+0.04 ± 0.02^**^
		4	+0.08 ± 0.02^**^			+0.08 ± 0.02^**^
V1 → mOFC	Happy-up	1	+0.05 ± 0.02^**^			+0.05 ± 0.02^**^
		4	+0.04 ± 0.02^**^			+0.04 ± 0.02^**^
	Happy-down	1	−0.00 ± 0.02			−0.00 ± 0.02
		4	+0.01 ± 0.02^*^			+0.01 ± 0.02^*^
	Fear-down	1	+0.12 ± 0.02^**^			+0.12 ± 0.02^**^
		4	+0.01 ± 0.02^*^			+0.01 ± 0.02^*^
	Fear-up	1	+0.11 ± 0.02^**^			+0.11 ± 0.02^**^
		4	+0.04 ± 0.02^**^			+0.04 ± 0.02^**^
FFA → Amy	Happy-up	1	+0.07 ± 0.03^**^	+0.16 ± 0.09^**^	+0.36 ± 0.14^**^	+0.59 ± 0.15^**^
		4	−0.01 ± 0.03	+0.56 ± 0.12^**^	+0.27 ± 0.17^**^	+0.83 ± 0.21^**^
	Happy-down	1	+0.10 ± 0.02^**^	−0.03 ± 0.13	+0.02 ± 0.14	+0.09 ± 0.19
		4	+0.03 ± 0.02^**^	+0.14 ± 0.15^*^	+0.02 ± 0.17	+0.19 ± 0.23^*^
	Fear-down	1	+0.01 ± 0.02	+0.55 ± 0.09^**^	+0.31 ± 0.13^**^	+0.86 ± 0.16^**^
		4	+0.04 ± 0.02^**^	+0.29 ± 0.10^**^	−0.01 ± 0.16	+0.32 ± 0.17^**^
	Fear-up	1	+0.04 ± 0.02^**^	+0.23 ± 0.13^**^	+0.11 ± 0.15^*^	+0.38 ± 0.19^**^
		4	+0.01 ± 0.02^*^	+0.04 ± 0.12	+0.19 ± 0.14^**^	+0.24 ± 0.19^**^
Amy → FFA	Happy-up	1	+0.13 ± 0.02^**^	+0.05 ± 0.04^**^	+0.01 ± 0.07	+0.18 ± 0.06^**^
		4	+0.04 ± 0.02^**^	+0.10 ± 0.09^**^	−0.02 ± 0.11	+0.12 ± 0.15^*^
	Happy-down	1	+0.12 ± 0.02^**^	−0.04 ± 0.14	+0.48 ± 0.16^**^	+0.56 ± 0.21^**^
		4	+0.06 ± 0.02^**^	+0.08 ± 0.15^*^	−0.03 ± 0.15	+0.11 ± 0.21
	Fear-down	1	+0.12 ± 0.02^**^	−0.02 ± 0.07	−0.02 ± 0.08	+0.08 ± 0.10^*^
		4	+0.06 ± 0.02^**^	+0.13 ± 0.08^**^	+0.04 ± 0.11	+0.23 ± 0.13^**^
	Fear-up	1	+0.06 ± 0.02^**^	−0.02 ± 0.12	−0.06 ± 0.14	−0.03 ± 0.18
		4	+0.04 ± 0.02^**^	+0.00 ± 0.13	+0.12 ± 0.15^*^	+0.17 ± 0.20^*^
Amy → mOFC	Happy-up	1	+0.03 ± 0.02^**^	+0.21 ± 0.04^**^	−0.14 ± 0.07^**^	+0.10 ± 0.07^**^
		4	−0.01 ± 0.02^*^	+0.15 ± 0.09^**^	−0.15 ± 0.11^**^	−0.01 ± 0.15
	Happy-down	1	+0.01 ± 0.02	+0.05 ± 0.13	−0.03 ± 0.14	+0.03 ± 0.19
		4	+0.04 ± 0.02^**^	+0.04 ± 0.14	+0.05 ± 0.15	+0.13 ± 0.21^*^
	Fear-down	1	+0.08 ± 0.02^**^	+0.03 ± 0.07	+0.24 ± 0.09^**^	+0.34 ± 0.11^**^
		4	+0.03 ± 0.02^**^	+0.09 ± 0.08^**^	+0.01 ± 0.11	+0.13 ± 0.13^*^
	Fear-up	1	+0.03 ± 0.02^**^	+0.01 ± 0.12	−0.07 ± 0.13	−0.02 ± 0.17
		4	+0.00 ± 0.02	+0.03 ± 0.13	+0.03 ± 0.14	+0.05 ± 0.20
mOFC → Amy	Happy-up	1	+0.13 ± 0.03^**^	+0.06 ± 0.10^*^	−0.03 ± 0.15	+0.15 ± 0.18^*^
		4	−0.03 ± 0.02^**^	+0.24 ± 0.12^**^	+0.02 ± 0.15	+0.22 ± 0.20^**^
	Happy-down	1	−0.02 ± 0.03^*^	+0.19 ± 0.15^**^	+0.14 ± 0.16^*^	+0.31 ± 0.23^**^
		4	+0.00 ± 0.02	+0.02 ± 0.16	+0.17 ± 0.17^*^	+0.20 ± 0.23^*^
	Fear-down	1	+0.02 ± 0.02^*^	−0.24 ± 0.12^**^	−0.16 ± 0.15^**^	−0.38 ± 0.19^**^
		4	−0.03 ± 0.02^**^	+0.01 ± 0.11	−0.06 ± 0.15	−0.08 ± 0.18
	Fear-up	1	+0.02 ± 0.02^*^	+0.13 ± 0.14^*^	−0.05 ± 0.16	+0.09 ± 0.21
		4	−0.01 ± 0.02	+0.11 ± 0.15^*^	−0.01 ± 0.17	+0.09 ± 0.22

Intrinsic connectivity [A], modulation by faces [B_1_] and intensity [B_2_], and total task-dependent connectivity [A + B_1_ + B_2_]. ^*^Significance at *p* < 0.05. ^**^Significance at *p* < 0.001.

Within groups significant changes for FFA and amygdala connectivity were observed in happy-up (M = 0.83 ± 0.21) vs. happy-down [M = 0.19 ± 0.23, *t*(15) = 8.30, *p* < 0.001], happy-up (M = 0.83 ± 0.21) vs. fear-down [M = 0.32 ± 0.17, *t*(15) = 7.50, *p* < 0.001], and happy-up (M = 0.83 ± 0.21) vs. fear-up [M = 0.25 ± 0.19, *t*(15) = 8.26, *p* < 0.001]. Whereas, the significant mOFC and amygdala connectivity changes were observed in the happy-up (M = 0.22 ± 0.19) vs. fear-down [M = −0.08 ± 0.19, *t*(15) = 4.48, *p* < 0.001], happy-down (M = 0.19 ± 0.23) vs. fear-down [M = −0.08 ± 0.18, *t*(15) =3.71, *p* < 0.001], and fear-down (M = 0.08 ± 0.18) vs. fear-up [M = 0.09 ± 0.22, *t*(15) = −2.35, *p* = 0.03].

### Psychometric changes

LMM analyses of psychometric measures revealed no significant effect of time, group, and interaction on PANAS and SDS scores in any group in the happy and fear conditions.

## Discussion

In the current study, we introduced an innovative neurofeedback design using dynamically adapting naturalistic face stimuli (happy and fearful faces) as a feedback signal coupled to the participant’s ongoing amygdala activity. Healthy participants were trained in four groups to upregulate or downregulate their amygdala explicitly by modulating the valence of the face stimulus. Participants in the happy condition were instructed to make the face happier either by upregulating (happy-up group, task-congruent) or downregulating (happy-down group, task-incongruent) their amygdala activity. Meanwhile, participants in the fear condition were instructed to reduce fearfulness of the face through amygdala downregulation (fear-down group, task-congruent) and through upregulation (fear-up group, task-incongruent). Thus, with such a versatile neurofeedback design, we investigated the effects of the stimulus (happy vs. fearful faces), regulation condition (upregulation vs. downregulation), and task congruency (task-congruent vs. incongruent) on learning success.

### Training-induced amygdala regulation

We assessed changes in the amygdala activity across four runs using LMM analysis which revealed a significant effect of run, mainly in the fear-down group (Figure 3). Higher amygdala activity was observed in all groups during the first training run which may contribute to the novelty of the emotionally salient facial stimulus (Balderston et al., 2011). Particularly, we observed a decrease in the amygdala activity in the fear-down group in the last two runs as compared to the first run. Decreasing amygdala activity in the fear-down group is congruent with the task instruction, but not in the fear-up group. It is possible that a task-congruent condition may have facilitated the perception of reward which might have encouraged the participant’s regulatory effort. Whereas, in the fear-up group, a conflict between the high-level goal of amygdala upregulation (by making the face less fearful) and intrinsic desire to perceive neutral or less intense faces as rewarding (by implicitly downregulating the amygdala) may have contributed to unsuccessful upregulation.

However, contrary to our hypothesis, we did not observe a significant increase in the amygdala BOLD signal in the happy-up group, where the task instructions were congruent. A possible explanation is a ceiling effect, as reported by Paret et al. (2018), where healthy participants could not achieve successful amygdala upregulation while exposed to aroused pictures.

### Habituation effects

Some of the observed effects, particularly in the fear conditions, could be attributed to amygdala habituation. Similar effects have been observed previously (Plichta et al., 2012; Geissberger et al., 2020), however, these studies did not address the internal regulatory mechanisms and goal-directed behavior of the participants. We propose that the amygdala is not a passive processor of emotional stimuli that becomes habituated when the stimuli are no longer novel. Instead, it is part of a complex network that is also influenced by the participant’s ongoing motivational goals, expectations, and other cognitive processes. We observed that the amygdala habituated in the fear-down and fear-up groups, but not in the happy-up and happy-down groups. This suggests that the participant’s goal-directed behavior and the valence of the stimulus influence potential amygdala habituation effects.

### Connectivity changes associated with the amygdala

We also observed amygdala effective connectivity changes using DCM that were specific to the experimental condition. We found that FFA to amygdala connectivity increased in the happy-up group and decreased in the fear-down group over four training runs. Consistent with our hypothesis, amygdala upregulation is associated with positive (i.e., facilitatory) and downregulation with negative (i.e., inhibitory) connectivity between the mOFC and the amygdala. This finding is supported by the previous effective connectivity studies suggesting that amygdala downregulation is mediated by the mOFC when participants view emotional faces (Sladky et al., 2015; Minkova et al., 2017). In the first run, the amygdala was strongly downregulated by the mOFC in the fear-down group as compared to the other groups. This downregulation effect persisted until the last run. It appears that during the first run most of the regulation occurs top-down via the mOFC, while in the last run, the bottom-up influence of the FFA is increased. While the present study cannot provide a definitive answer for the underlying mechanisms, we can speculate that upregulating (happy-up) or downregulating (fear-down) the influence of the external stimulus would be more efficient than prefrontal regulation, both neuronally (i.e., because of long-range connections) and psychologically (i.e., because of cognitive effort). Conceptualizing neurofeedback learning as a metabolically efficient adaptation of hierarchical regulation loops contrasts the view that the amygdala is a statically mapped, stimulus-driven system. Instead, viewing the amygdala as a dynamic control circuit would be in line with an active inference interpretation of amygdala function, where amygdala communication occurs via flexible perceptual predictions and resulting prediction errors (Sladky et al., 2023).

Reduced effective connectivity between the OFC and the amygdala has also been observed during increased cognitive workload (Minkova et al., 2017) and in patients with social anxiety disorder (Sladky et al., 2015). The orbitofrontal cortex appears to play a central role in social anhedonia (Germine et al., 2011). Consistent with this model, MDD patients showed a decreased amygdala upregulation by the orbitofrontal cortex when exposed to happy faces as compared to healthy controls (de Almeida et al., 2009). In our study, significant affect-specific changes were observed only in the task-congruent groups such as happy-up and fear-down, supporting an innate tendency of neural processes to increase positive and decrease negative affectivity. This may suggest an adaptive learning process guided by mOFC activity during the training. It is highly plausible that the connectivity between mOFC and amygdala is rapidly updated during task performance, as mOFC is thought to be involved in regulation mechanisms that enable adaptive behavior (Lichtenberg et al., 2017).

### Behavioral changes

We did not observe significant changes in the psychometric questionnaire scores of the participants in any of the groups. As this feasibility study was conducted in healthy participants to test the efficacy of the naturalistic feedback approach, it is less likely that the training will produce lasting or strong changes in the affectivity in participants without mood impairments. Therefore, the plausibility of this novel training approach in reducing negative mood and anxiety symptoms should be evaluated in a clinical sample such as MDD and AD patients. In addition, the pragmatic utility of the amygdala dynamic face feedback training for improving affective responses in real-life environment needs to be investigated in future studies.

### Applications of the naturalistic closed-loop neurofeedback

This innovative approach allowed participants to train self-regulation of their amygdala by changing the emotional expression of facial stimuli. Previous rtfMRI-NF studies have reported successful amygdala downregulation in healthy participants while viewing negative emotional faces (Brühl et al., 2014) or aversive situations (Sarkheil et al., 2015; Paret et al., 2016; Herwig et al., 2018), and in clinical populations such as patients suffering with PTSD (Nicholson et al., 2016) and bipolar disorder (Zaehringer et al., 2020). Amygdala upregulation was also achieved through positive autobiographical memory recall in healthy subjects (Zotev et al., 2016; Hellrung et al., 2018) and MDD patients (Young et al., 2017). Moreover, Paret et al. (2018) demonstrated concurrent upregulation and downregulation of the amygdala in healthy female participants while viewing emotional images. Our new experimental setup extends these previous findings by providing an ecologically valid, naturalistic face feedback training that could be a versatile neuroscience research tool and a novel experimental therapy.

Compared to the abstract feedback representations used in these studies, the use of emotional faces is highly relevant to studies investigating how we perceive emotions and regulate subsequent neural responses. Social cognition involves the processing of verbal and nonverbal social cues by identifying, perceiving, and interpreting other people’s behaviors, intentions, feelings, and beliefs (Frith and Frith, 2006). Faces are an important source of information relevant to the perception of the mental state of others (Weightman et al., 2014). As a result, humans are highly trained to discriminate static (e.g., identity, gender, age) and dynamic features (e.g., gaze direction and emotional expression) of other peoples’ faces (Bruce and Young, 1986). While static features are processed in the fusiform gyrus (Haxby et al., 2000), their dynamicity is processed in the superior temporal sulcus (STS) with the amygdala and insula for processing of emotional expressions (Haxby et al., 2000). Thus, using a facial feedback with dynamically adapting affect is more realistic, socially rewarding and increases the sense of agency as compared to the symbolic feedback representation. Furthermore, it reduces the cognitive load associated with participants’ explicit regulatory efforts by preventing dual-task interference between visual cues and feedback signal (Ihssen et al., 2017; Sitaram et al., 2017).

Naturalistic face feedback could lead to more rewarding social interactions which is specifically relevant for autism spectrum disorders (Dichter, 2018), SAD (Sladky et al., 2012), and addiction disorders, where the hedonic value of social rewards is reduced. Regarding the latter, most studies address the “wanting” component of addiction (e.g., compulsive thoughts; Koob and Le Moal, 1997; Sulzer et al., 2013; Kirschner et al., 2018), which is thought to be mediated by the dopaminergic reward network. Amygdala neurofeedback using naturalistic social rewards could be a complementary method to address the “liking” component of hedonic experience (Robinson and Berridge, 2000; Haugg et al., 2022). Thus, presenting social feedback in the form of emotional faces may be beneficial in training healthy individuals and psychiatric patients to improve social cognition.

Given the variety of methodological and psychological questions that can be addressed with such an adaptive facial feedback design, its use is not solely restricted to training a single brain region such as the amygdala. It can be tailored to address other affect processing brain regions like the anterior cingulate cortex (ACC; Mathiak et al., 2010, 2015) or even functional networks (Ramot et al., 2017; Koush et al., 2017b; Zich et al., 2020; Taylor et al., 2022) to regulate neural responses that govern emphatic behavior and social cognition. Thus, dynamic emotional faces serve as suitable naturalistic feedback stimuli for shaping clinically relevant amygdala activity.

### Limitations

This feasibility study has several limitations. We used a high-resolution anatomical mask to define the region of interest (ROI), i.e., bilateral amygdalae (Tyszka and Pauli, 2016). The amygdala is a small subcortical structure that is functionally dynamic and anatomically diverse (Tyszka and Pauli, 2016; Wang et al., 2017), so using a predefined mask to generate the feedback signal could be a limitation. Comparatively, a functional localizer task may have higher accuracy in defining participant-specific ROI that facilitates neurofeedback learning, but there is currently no supporting evidence (Haugg et al., 2021). A more refined ROI definition based on amygdala sub-nuclei may improve the quality of the neurofeedback signal which can be achieved with ultra-high field imaging at 7-Tesla fMRI, which provides a higher signal-to-noise ratio, improving spatial specificity in the subcortex (Hahn et al., 2013; Sladky et al., 2018).

Another limitation could be a restricted field of view (temporal lobe, visual cortex, and mOFC), which hindered understanding the potential influence of affect processing brain regions other than the amygdala, specifically, the cortical areas and other face processing areas such as STS and occipital face area (OFA). We restricted our data acquisition to the ventral brain because we wanted to investigate the involvement of the FFA and mOFC in addition to the amygdala. While prefrontal regions such as dorsolateral prefrontal cortex (DLPFC) play an important role in reappraisal mechanisms (Morawetz et al., 2016), we previously identified the mOFC as the most relevant causal influence on amygdala regulation (Sladky et al., 2022). However, other PFC regions engaged through the participants’ explicit cognitive regulatory efforts and emotion perception regions like STS which is responsible for social cognition (Direito et al., 2021) are highly relevant and require further exploration. Future studies using faster acquisition methods such as multiband EPI sequences and higher field strengths will allow for better brain coverage without compromising the image resolution.

The main limiting factor could be the short duration of the neurofeedback training. In this proof-of-concept study, participants completed only four training runs within a single session of neurofeedback training. It is possible that such short-duration training is not enough to induce learning and subsequent behavioral changes. Hence, it is difficult to make any causal inferences regarding the effectiveness of the novel naturalistic face neurofeedback design. Currently, there is no consensus on the optimal number of neurofeedback runs or sessions required to achieve desired neural and behavioral changes in healthy and in clinical populations (Paret et al., 2019). Furthermore, recent evidence suggests that patients are more successful than healthy participants at learning self-regulation of brain activity (Haugg et al., 2021). Therefore, enrolling more participants in intensive longitudinal neurofeedback training and testing this novel approach in psychiatric disorders may lead to clinically relevant neural and behavioral changes.

Another main limitations of the study are the lack of a control group and the no-feedback practice (i.e., baseline) and transfer runs. The inclusion of a control condition is necessary to improve the specificity of the training and to rule out non-specific factors influencing the regulation success (Sorger et al., 2019). Thus, without a control group in the current study, it is difficult to ascertain causal effects of the naturalistic face neurofeedback training. A recent meta-analysis has reported a positive correlation between the learning success and pre-training practice run without feedback (Haugg et al., 2021). While a practice run may enhance the effectiveness of the chosen regulation strategies during the actual training, a post-training no-feedback run, or a transfer run is a prerequisite for improving the transferability of the learned behavior to real life. These aspects of neurofeedback training are not addressed in the current study.

The type of adaptive emotional face feedback used in the current study may pose a challenge in determining whether the observed changes in amygdala activity are attributed to participants’ conscious regulatory efforts based on the feedback they receive (feedback-driven) or are solely a response to the facial stimuli (stimulus-driven). This is because face feedback not only represents the brain’s response to external stimuli (e.g., dynamic facial emotions) but also simultaneously influences the person’s ability to regulate brain activity (Direito et al., 2019). This distinction is crucial for ascertaining the effectiveness of neurofeedback training. However, disentangling these two aspects is methodologically complex given the small sample size and beyond the scope of the current study. Larger and more diverse study samples (e.g., including clinical populations) might allow investigating the individual differences in emotion and amygdala regulation success at the level of effective connectivity differences. Furthermore, follow-up studies could employ specifically designed transfer runs or sham feedback conditions. Future neurofeedback studies using an adaptive feedback interface should aim to investigate the stimulus-driven and self-regulation-driven changes in brain activity.

The unavailability of the data on subjective performance during the training could be another confound. Participants included in the study were given the freedom to use emotion regulation strategies of their choice and were briefed about the amygdala neurofeedback and subsequent training goals (i.e., to make the face happier or less fearful). They were asked to verbalize the mental strategy that they intended to use before each neurofeedback run to avoid mind-wandering and rumination during the training. The most common strategies reported by the participants to make the face happier were mentally telling a joke, imagining positive memories, imagining tickling a person etc., whereas, to reduce the fearfulness of the face, calming the person down, imagining walking by the lake, hugging a person etc., were used. However, we did not include the individual regulation strategies used by the participants during the training in our analysis as we did not perform a structured qualitative interview. The use of appropriate and effective mental strategies may be central to the cognitive learning and may have an influence on neural processes and subsequent behavior, especially, in patients with psychiatric disorders (Mac Duffie et al., 2018; Herwig et al., 2019). Hence, future neurofeedback studies should consider including subjective data to address regulatory success.

## Conclusion

In conclusion, the results of this proof-of-concept study in healthy participants have demonstrated that the dynamic emotional face neurofeedback design offers a powerful experimental approach for investigating complex interactions between stimuli, regulation conditions, and adaptive feedback signals to efficiently shape brain activity. Although the present study did not confirm the clinical efficacy of closed-loop naturalistic face neurofeedback, the literature has demonstrated the therapeutic benefits of interactive feedback interfaces such as dynamic face stimuli in clinical settings (Direito et al., 2021). In this context, such a novel naturalistic face feedback design could be an extension of currently developing adaptive neurofeedback protocols for affective and other psychiatric disorders.

## Data availability statement

The raw data supporting the conclusions of this article will be made available by the authors on request, without undue reservation.

## Ethics statement

The studies involving humans were approved by Kantonale Ethikkommission Zürich. The studies were conducted in accordance with the local legislation and institutional requirements. The participants provided their written informed consent to participate in this study.

## Author contributions

AW: Data curation, Formal analysis, Project administration, Writing – original draft, Methodology. AH: Data curation, Writing – review & editing. NF: Data curation, Writing – review & editing. YK: Software, Writing – review & editing. DW: Software, Writing – review & editing. AB: Conceptualization, Methodology, Writing – review & editing. PS: Software, Writing – review & editing. FS: Conceptualization, Funding acquisition, Investigation, Methodology, Resources, Supervision, Validation, Writing – review & editing. RS: Conceptualization, Data curation, Formal analysis, Funding acquisition, Investigation, Methodology, Project administration, Supervision, Writing – original draft, Writing – review & editing.

## Glossary

**Table:** 

WHO	World Health Organization
MDD	Major depressive disorder
AD	Anxiety disorders
PTSD	Posttraumatic stress disorder
SAD	Social anxiety disorder
fMRI	Functional magnetic resonance imaging
rtfMRI-NF	Real-time fMRI neurofeedback
TR	Repetition time
TE	Echo-time
EPI	Echo planar imaging
FOV	Field of view
ROI	Region of interest
FWHM	Full width at half maximum
MNI	Montreal Neurologic Institute
GLM	General linear model
SPM	Statistical parametric mapping
DCM	Dynamic causal modeling
BMA	Bayesian model averaging
cHRF	Canonical hemodynamic response function
BOLD	Blood oxygenation level dependent
FFA	Fusiform face area
mOFC	Medial orbitofrontal cortex
ACC	Anterior cingulate cortex
DLPFC	Dorsolateral prefrontal cortex
PANAS	Positive and negative affect schedule
SDS	Self-rating depression scale
ANOVA	Analysis of variance
STS	Superior temporal sulcus

## Footnotes

^1^ https://github.com/alyssaq/face_morpher

^2^ https://github.com/alyssaq/facemorpher
