# Supplementary material for: Facing emotions: real-time fMRI-based neurofeedback using dynamic emotional faces to modulate amygdala activity
Source: Front Neurosci. 2024 Jan 11;17:1286665. doi: 10.3389/fnins.2023.1286665 (PMC10808718; doi:10.3389/fnins.2023.1286665)
Supplement: Supplementary file 1 [file Data_Sheet_1.PDF]

CRED-nf checklist summary output

1. Pre-experiment
    a. This experiment was not preregistered
    b. The manuscript does not describe the sampling plan or justify the sample size used
2. Control groups
    a. This experiment did not include a control group or control condition
    b. NA: A double-blind was not appropriate for this experiment
    c. Blinding of those who rate the outcome and those who analyse the data:
        - Those who rated the outcome were not blind to group assignment
        - Those who analysed the data were not blind to group assignment
    d. No measures were taken to examine whether participants and experimenters remained blind
    e. NA: This is not a clinical efficacy study
3. Control measures
    a. Training-induced changes in affectivity were assessed before and after the neurofeedback session using the self-rated Positive And Negative Affect Schedule (PANAS) and the Self-rating Depression Scale (SDS).
    b. The manuscript does not report whether strategies were provided
    c. The most common strategies reported by the participants to make the face happier were mentally telling a joke, imagining positive memories, imagining tickling a person etc., whereas, to reduce the fearfulness of the face, calming the person down, imagining walking by the lake, hugging a person etc., were used
    d. Online rt-fMRI data analysis and neurofeedback signal calculation were performed using OpenNFT (Koush et al., 2017). OpenNFT's default online preprocessing pipeline was used which comprised real-time realignment for motion correction and spatial smoothing (Gaussian kernel, 5mm FWHM). Temporal data processing included spike removal using a Kalman filter, drift removal using a cumulative GLM, a first-order autoregressive model AR(1) to account for serial correlations, and OpenNFT's default dynamical range scaling.
    e. Condition and group effects for artifacts were not measured, or not reported in the manuscript
4. Feedback specifications
    a. The estimation and presentation of the feedback signal was achieved using the Open NeuroFeedback Training (OpenNFT) software, an open-source neurofeedback framework implemented using Python and Matlab (Koush et al., 2017a). The feedback was scaled to the normalized amygdala time-series using OpenNFT's default dynamic range to estimate the maximum and minimum limits of the scaling (Koush et al., 2017a) by using the average of the 5% highest and lowest signal intensities observed so far. The preprocessed amygdala signal was then mapped to the intensity of the emotional expression ranging between the lowest and the highest valence of the face stimulus that served as the feedback signal.
    b. The manuscript does not report or justify the reinforcement schedule
    c. The naturalistic face stimuli consisted of human faces of thirty Caucasian models (15 females) from the Radboud Face Database depicting neutral, fearful, and happy emotions (Langner et al., 2010). A face morphing algorithm developed in Python was used to create dynamic emotional faces with gradually changing facial expressions in thirty steps (https://github.com/alyssaq/facemorpher), such that 0 corresponded to the lowest valence or neutral emotion (0%) and 30 to the highest valence (100%), i.e. fearful or happy (smiling) expressions. The intensity of the emotional valence

was coupled to the average BOLD signal of the participant's bilateral amygdalae. All brain activity variable(s) and/or contrasts used for feedback, as displayed to experimental participants were not collected or are not reported in the manuscript

   d. The estimation and presentation of the feedback signal was achieved using the Open NeuroFeedback Training (OpenNFT) software, an open-source neurofeedback framework implemented using Python and Matlab (Koush et al., 2017a). The feedback was scaled to the normalized amygdala time-series using OpenNFT's default dynamic range to estimate the maximum and minimum limits of the scaling (Koush et al., 2017a) by using the average of the 5% highest and lowest signal intensities observed so far. The preprocessed amygdala signal was then mapped to the intensity of the emotional expression ranging between the lowest and the highest valence of the face stimulus that served as the feedback signal.

5. Outcome measures - Brain
   a. With such a versatile and comprehensive feedback design, we hypothesized that healthy participants would learn to up- and downregulate their amygdala activity in the task-congruent groups, i.e., happy-up and fear-down, respectively, as compared to the respective task-incongruent groups (happy-down and fear-up)
   b. Neural correlates of neurofeedback training were examined using LMM analysis by modeling run (1-4) and group (congruent and incongruent) as the fixed effects and a random intercept for participant separately in the happy and fear conditions. We observed a significant effect of run [$F(3,90) = 5.28$, $p = .0002$] in the fear condition with decreased amygdala activity in the third run [$\beta = -0.34$, $SE = 0.11$, $t(90) = -3.04$, $p = .003$], and the fourth run [$\beta = -0.34$, $SE = 0.11$, $t(90) = -3.02$, $p = .003$] compared to the first neurofeedback run in the fear-down group. We did not observe a significant effect of group (fear-down, fear-up) or the interaction between fixed effects, i.e., run x group. In the happy condition, LMM analyses revealed no significant effect of run, group (happy-up, happy-down), or their interaction. Amygdala activity changes over four training runs in all groups are depicted in Figure 4.
   c. The manuscript does not statistically compare the experimental condition/group to the control condition(s)/group(s)
6. Outcome measures - Behaviour
   a. LMM analyses of psychometric measures revealed no significant effect of time, group, and interaction on PANAS and SDS scores in any group in the happy and fear conditions.
   b. This manuscript does not compare regulation success and behavioural outcomes
7. Data storage
   a. No additional documents related to the materials, analysis scripts, code, raw data, or final values are available for this manuscript
